# Trial-based clinical and economic analyses: the unhelpful quest for conformity

**DOI:** 10.1186/1745-6215-14-421

**Published:** 2013-12-05

**Authors:** David GT Whitehurst, Stirling Bryan

**Affiliations:** 1 Faculty of Health Sciences, Blusson Hall 10504, Simon Fraser University, 8888 University Drive, Burnaby, British Columbia V5A 1S6, Canada; 2 Centre for Clinical Epidemiology & Evaluation, Vancouver Coastal Health Research Institute, 7th Floor 828 West 10th Avenue, Vancouver, British Columbia V5Z 1M9, Canada; 3 School of Population & Public Health, University of British Columbia, 2206 East Mall, Vancouver, British Columbia V6T 1Z3, Canada

**Keywords:** Randomized controlled trial, Economic evaluation, Outcomes measurement, Cost-utility analysis, Scientific paradigms, Estimation, Inference

## Abstract

Where there is conformity across the findings, interpretation and implications of 'clinical’ and 'economic’ research, there is limited cause for concern. However, there is often unease when apparent contradictory conclusions are drawn from the same study. Given the ever increasing role for economic evaluation in healthcare decision making, this commentary challenges the necessity of compatibility between clinical and economic evaluation.

## Background

With many countries requiring evidence of cost-effectiveness prior to passing judgment on the value of a medical intervention, it is inevitable that clinical and economic evidence will be considered concurrently by policy makers [1,2]. Such an approach enables decision makers to reflect on scientific and social value judgments (addressing both equity and efficiency) when allocating scarce resources [3]. In circumstances where there is conformity across the findings and implications of clinical and economic research, few concerns are raised. However, there is often unease when apparent contradictory conclusions are drawn within a study, either because the clinical research shows benefit but the economics indicates that a therapeutically beneficial intervention is not cost-effective [4,5], or the clinical research shows little benefit but the economics reveals evidence of cost-effectiveness for one of the interventions being evaluated [6]. For example, a recent study reported that a lifestyle program for adults at risk for type 2 diabetes and/or cardiovascular disease was not more effective in reducing these risks when compared to general health brochures, although the intervention had a high probability of being cost-effective [7].

The former situation is a reflection of the unavoidable need for healthcare decision-making within the constraints of a finite budget; sometimes, health benefits attributable to an intervention may not be sufficient to warrant associated increases in expenditure. Unease in the latter situation relates, primarily, to the different methods of analysis adopted in clinical and economic evaluation. Differing opinions regarding the role of economic evaluation alongside randomized controlled trials (RCTs) with indeterminate clinical findings can result in difficulties/delays in publishing economic evaluation results, irrespective of whether concerns are expressed within the study team or during the peer-review process. In this commentary, we challenge the necessity of compatibility between clinical and economic trial-based research.

## Clinical and economic evaluation: distinct scientific paradigms

Thomas Kuhn, the American physicist and philosopher, wrote extensively on the history of science and, in particular, on the notion of paradigms in science. Describing a paradigm as 'the entire constellation of beliefs, values, techniques, and so on shared by the members of a given community’ [8], Kuhn’s definition provides an ideal backdrop to revisit current, widely-practiced clinical and economic evaluative frameworks, that is, pragmatic RCTs and trial-based cost-effectiveness analyses.

There are stark differences between clinical and economic evaluation with respect to the purpose of research ('belief’) and the adopted analytic methodologies ('technique’). Differences in belief, in this context, exist in the respective objectives of clinical intervention studies and economic evaluation. The pragmatic RCT is conducted to quantify the magnitude of some factor(s) of interest, providing an estimate of effectiveness for an intervention compared to an appropriate control group. The purpose is to determine the ability of an intervention to improve patient-level outcome(s) (or, in the case of noninferiority trials, to test whether an intervention is not unacceptably worse than a current procedure or treatment) and the contribution of the research lies in the increased evidence base for clinicians and researchers. Alternatively, the purpose of cost-effectiveness analysis is to provide decision-makers with evidence on the value of an intervention, reflecting efficiency and equity [3]. This evidence is then considered against competing claims for healthcare resources across a multitude of medical conditions.

Despite fundamental differences between clinical and economic evaluation, the distinction is somewhat blurred with respect to analytic methodologies, despite the availability of research methods and reporting guidelines for trial-based economic analyses [9]. As an illustration, many readers will have read or heard (or said) comments such as, 'economic analysis is only important if there is evidence of clinical benefits.’ In our opinion, this is wholly inappropriate as a starting point for consideration of cost-effectiveness. Demonstrable clinical benefit is not a prerequisite for economic evaluation; large clinical benefits may be too expensive, small clinical benefits may be cost-effective and, dare we say, health decrements may be associated with sufficient cost savings to warrant an intervention becoming part of routine care. We use the words 'large' and 'small', as opposed to 'significant' and 'non-significant', to emphasize the difference in analytic focus between conventional economic evaluation (estimation) and clinical evaluation (inference), which has been discussed elsewhere [9-12]. We return to the questionable use of inferential statistics for economic evaluation in the following section.

Given the different research objectives of RCTs and economic evaluations, it is not surprising that differences exist with regard to the measurement of patient-level benefit ('value’). Clinical relevance is the key determinant when selecting the primary outcome in a clinical trial [13]; the chosen outcome measure is required to measure patients’ response to treatment, often focusing on a condition-specific measure of response that is meaningful to the clinical community, with the results being interpretable at the individual patient level. However, the relevance of the chosen clinical outcome measure is likely negligible when exploring the 'economic’ consequences of a particular intervention. For example, assessment of cost-effectiveness within a cost-utility framework brings together two components: costs (defined by the perspective of the analysis) and quality-adjusted life years (QALYs). The standard approach for estimating QALYs provides for the incorporation of societal health state values through the use of preference-based health-related quality of life (HRQoL) measures [1,2].

The results of an economic evaluation only have meaning in a comparative sense. Although clinical effectiveness can be explored within the boundaries of a single study, value judgments regarding efficiency and equity require consideration of other demands on healthcare resources. The pursuit of this broader, societal objective - albeit with patient-level data - is a reflection that society comprises individuals, and all individuals are potential patients.

## Confusing analytic conciliation

The health economics research community has provided its own contribution to the evaluative confusion. In particular, with respect to the measurement of health benefit, studies published in recent years have subjected preference-based HRQoL measures to concepts that are fundamentally inferential in nature; namely, the minimally important difference (MID) and the noninferiority margin [14-16].

The MID is 'the smallest difference in score in the domain of interest which patients perceive as beneficial and which would mandate, in the absence of troublesome side effects and excessive cost, a change in the patient’s management’ [17]. Although the concept of the MID is well accepted in clinical research, we believe there is some concern regarding its application for cost-effectiveness research (despite a number of studies having estimated MIDs for preference-based HRQoL instruments [15,16]). MIDs, however defined, are specific to a single outcome measure and are interpreted as being applicable at a patient-level, meaning that the MID concept is too narrow to be meaningful in economic evaluation. Cost-effectiveness estimation requires the simultaneous consideration of costs and effects. The magnitude of a cost (effect) difference, viewed in isolation, holds limited value until combined with the respective difference in effects (costs). Just because it is possible to construct a minimally important difference for preference-based measures does not mean that it is useful.

Similar issues arise in relation to noninferiority trials [14]. Guidelines for the conduct of economic evaluation alongside such trials state that acceptable differences in costs and effects must be defined *a priori* in order to explore non-inferior or equivalent cost-effectiveness of study treatments. However, these requirements take no account of the key analytic focus of economic evaluation: estimation of the joint density of cost and effect differences [11]. Determining whether a cost difference or QALY difference is 'acceptable’ is not the role of the analyst. In a trial-based economic evaluation, irrespective of the clinical findings or RCT design (superiority, noninferiority, or equivalence), an analyst should focus on estimating cost and effect differences and quantifying the likelihood that an intervention is cost-effective [9,12].

## Conclusions

There is a need to understand better the fundamental differences in the questions being addressed by clinical and economic evaluation performed alongside RCTs, and for all researchers to be comfortable with apparently discordant findings. It is our hope that this commentary will provide clarity, or spark further debate, and afford a reference point for future discussions about 'incompatible’ RCT findings.

## Abbreviations

HRQoL: Health-related quality of life; MID: Minimally important difference; QALY: Quality-adjusted life year; RCT: Randomized controlled trial.

## Competing interests

The authors declare that they have no competing interests.

## Authors’ contributions

Both authors were involved in the conception and framing of the Commentary, and have read and approved the final manuscript. DGTW wrote the first draft and subsequent revisions. SB was involved in review and critical revision of the content prior to submission.

## Authors’ information

This paper draws on the experiences of both authors in the field of economic evaluation, covering methodological and applied research projects across a range of clinical specialties. More specifically, the conception of this Commentary relates to numerous conversations with colleagues about the distinctions between clinical and economic evaluation, and the frequent need to appease peer reviewers’ concerns about apparent mixed messages within a single study.
